# Psychological and religious predictors of help seeking attitudes among university students under perceived stress

**DOI:** 10.1038/s41598-026-36332-5

**Published:** 2026-01-27

**Authors:** Diem-Ngan Pham-Ngoc, Phuoc-Thien Nguyen, Nhu-Quynh Nguyen-Thi, Duc-Huy Nguyen, Cao-Dat Vo, Thien Tat Do, Cyrus Su Hui Ho, Vinh-Long Tran-Chi

**Affiliations:** 1 Faculty of Psychology, Ho Chi Minh City University of Education, 280 An Duong Vuong Street, Cho Quan Ward, Ho Chi Minh City, 700000 Vietnam; 2 Faculty of Business Administration, Ho Chi Minh City University of Economics and Finance, Ho Chi Minh City, 70000 Vietnam; 3 Department of Psychological Medicine, National University of Singapore, Singapore, 119007 Singapore; 4 Department of Psychological Medicine, National University Hospital, Singapore, 119007 Singapore; 5 University of Health Sciences, Vietnam National University, Ho Chi Minh City, 700000 Vietnam; 6 Vietnam National University, Ho Chi Minh City, 700000 Vietnam

**Keywords:** Perceived stress, Religious coping, Resilience, Professional help-seeking attitudes, University students, PLS-SEM, Public health, Population screening

## Abstract

This study aimed to examine how perceived stress, religious coping (both positive and negative), and resilience relate to Vietnamese university students’ attitudes toward seeking professional psychological help. It also investigated the mediating roles of religious coping and resilience in these relationships. Cross-sectional quantitative design using structural equation modeling (PLS-SEM). Data were collected online between November and December 2024 from students at multiple universities across Vietnam, coordinated by the Faculty of Psychology, Ho Chi Minh City University of Education. A total of 416 undergraduate students (263 females, 153 males; aged 18–25 years) completed the online survey. The sample included diverse academic majors, religious affiliations, and sexual orientations. Data were collected using the Socio-demographic Questionnaire, the Perceived Stress Scale (PSS-10), Brief Religious Coping Scale (Brief RCOPE), Brief Resilience Scale (BRS), and the Attitude Toward Seeking Professional Psychological Help Scale – Short Form (ATSPPH-SF). Perceived stress was positively associated with both positive and negative religious coping, and with greater openness toward professional help-seeking, but negatively related to resilience. Positive religious coping was a significant predictor of more favorable help-seeking attitudes and partially mediated the link between perceived stress and these attitudes. However, negative religious coping and resilience were not significantly associated with help-seeking attitudes and did not function as mediators. Subgroup analyses showed that LGBTQ + students were characterized by elevated stress and greater use of both positive and negative religious coping, whereas students with self-harm histories displayed higher stress and positive religious coping but lower resilience and help-seeking openness. Findings emphasize the dual role of religious coping as both a protective and maladaptive strategy in stressful situations. While positive coping enhances openness to help-seeking, resilience may act more as an internal resource than a motivator for external support. These results underscore the need for culturally sensitive interventions in universities that address not only psychological factors but also religious and social norms influencing mental health behavior.

## Introduction

Mental health concerns among university students have emerged as a critical public health issue worldwide, compelling educators and mental health professionals to develop responsive interventions within complex and rapidly evolving academic environments^1^. These challenges are especially pressing in low- and middle-income countries, where university-based mental health services remain limited or underdeveloped.

In Vietnam, although awareness of mental health has grown in recent years, it remains deeply influenced by collectivist cultural norms and traditional belief systems that frame psychological distress in distinctive ways^2^. Specifically, within the Vietnamese sociocultural context, Buddhism and indigenous folk beliefs place a strong emphasis on family cohesion and social conformity. This worldview often results in the internalization of mental health difficulties as familial or personal matters rather than as conditions warranting professional intervention^2^.

Common symptoms among university students—such as depression, examination-related anxiety, and sleep disturbances—contribute significantly to their psychological burden^1^. Yet despite this high prevalence, professional psychological help-seeking remains strikingly low^3^.

Several psychosocial barriers impede students from engaging in formal mental health services. These include low emotional competence, an overreliance on self-management, inadequate mental health literacy, and a strong desire to maintain social normalcy—all of which are compounded by internalized stigma and structural constraints such as cost and time^4–7^. Consequently, students tend to favor informal support networks, namely, friends and family, over professional services^8^.

Empirical studies in Vietnam have substantiated these barriers. For instance, Tran-Chi et al.^9^ found that among 478 Vietnamese undergraduates, COVID-19-related stress significantly mediated the link between self-concealment and negative attitudes toward seeking professional psychological help—particularly among students living alone^9^. Similarly, Nguyen et al.^10^ reported that while psychology majors in Hanoi demonstrated more positive help-seeking attitudes and lower public stigma compared to students from other majors, overall utilization of mental health services remained suboptimal^10^. A large-scale study at Can Tho, Vietnam further revealed that 69.5% of students reported experiencing stress, yet only 61.8% had received psychological advice and just 15.9% accessed formal consultancy services, despite an expressed demand exceeding 78%^11^. These findings collectively highlight persistent sociocultural and structural barriers to help-seeking and emphasize the urgent need for culturally informed mental health promotion strategies within Vietnamese higher education institutions.

While the roles of stigma, literacy, and structural barriers have been relatively well-documented, less attention has been paid to culturally embedded coping strategies—particularly religious coping, which may significantly influence help-seeking attitudes and behavior in the Vietnamese context.

Among the culturally embedded coping strategies available, religious coping represents a salient psychological mechanism through which individuals seek to manage distress. This construct encompasses a range of cognitive, emotional, and behavioral strategies rooted in religious or spiritual belief systems^12^. Positive religious coping involves constructive spiritual engagement—such as seeking divine support, reframing suffering, or finding existential meaning—whereas negative religious coping reflects spiritual discontent, moral guilt, or beliefs in divine punishment^13^. Empirical research has shown that religious coping not only moderates the association between stigma and help-seeking but also significantly correlates with psychological outcomes^14,15^. Resilience, the capacity to adapt positively in the face of adversity, has likewise been recognized as a crucial psychological resource that buffers the effects of academic and personal stressors^16,17^. Theoretical models increasingly suggest that both religious coping and resilience influence stress appraisals and subsequent coping strategies, particularly in collectivist cultural settings, where spiritual and community values are central to identity formation and help-seeking behavior^18^.

Grounded in Lazarus and Folkman’s^19^ Stress and Coping Theory^19^, the present study conceptualizes perceived stress as a psychological antecedent that initiates both cognitive and behavioral coping processes. Within this theoretical framework, stress appraisal activates coping responses that are influenced by sociocultural and spiritual belief systems. In the Vietnamese context, these responses are often intertwined with religious and moral worldviews that guide how individuals interpret and manage stress.

Positive and negative religious coping represent two divergent pathways within this stress–coping process. Positive religious coping reflects adaptive meaning-making, emotional regulation, and reliance on transcendent support, which may enhance resilience and openness to external psychological assistance. In contrast, negative religious coping involves spiritual discontent or punitive appraisals that can amplify distress and inhibit help-seeking behaviors. Resilience, conceptualized as an adaptive capacity to recover from stress, functions both as an outcome of coping processes and as a psychological mediator influencing subsequent attitudes and behaviors.

According to Lazarus and Folkman’s^19^ Stress and Coping Theory, psychological stress arises when individuals appraise environmental demands as exceeding their coping resources, prompting both cognitive and behavioral efforts to restore equilibrium. Within collectivist and religiously influenced societies such as Vietnam, these appraisal and coping processes are deeply embedded in cultural and spiritual frameworks^20,21^. Religious engagement—such as temple or church attendance, meditation, ancestral veneration, or participation in local spiritual practices—reinforces values of gratitude, filial duty, and emotional restraint that are central to moral education and self-regulation among Vietnamese youth^22^. For Vietnamese undergraduates, religious coping represents a culturally endorsed way to manage stress and sustain moral identity within a religious landscape encompassing Buddhism, Christianity (both Catholic and Protestant denominations), Caodaism, and Islam^23^.

Despite its relevance, little is known about how religious coping is practiced among Vietnamese university students in specific institutional and regional contexts. Unlike many countries where student counseling centers are integrated into campus life, Vietnamese universities—especially those outside major cities—often lack sufficient resources for psychological support.

To the best of our knowledge, no prior study has simultaneously examined perceived stress, religious coping (both positive and negative), resilience, and attitudes toward seeking professional psychological help within the Vietnamese sociocultural context. The present research, therefore, aims to address this gap by developing and empirically testing a multivariate path model that integrates these constructs into a single analytical framework. Specifically, we investigate both the direct and indirect (mediated) pathways through which perceived stress, religious coping, and resilience shape university students’ attitudes toward professional psychological help-seeking. By elucidating the underlying psychological mechanisms of help-seeking behavior among Vietnamese undergraduates, this study seeks to provide evidence-based insights to inform culturally sensitive mental health interventions in higher education settings.

Accordingly, we propose the following hypotheses:

Perceived Stress and Religious Coping

- H1a: Perceived stress will be positively associated with positive religious coping.
- H1b: Perceived stress will be positively associated with negative religious coping.
- H2: Perceived stress will be negatively associated with resilience.
- H3: Perceived stress will be positively associated with attitudes toward professional psychological help-seeking.

Religious Coping and Help-Seeking Attitudes

- H4a: Positive religious coping will be positively associated with attitudes toward professional psychological help.
- H4b: Negative religious coping will be negatively associated with attitudes toward professional psychological help.

Resilience and Help-Seeking Attitudes

- H5: Resilience will be positively associated with attitudes toward professional psychological help.

Interrelationship between Religious Coping and Resilience

- H6a: Positive religious coping will be positively associated with resilience.
- H6b: Negative religious coping will be negatively associated with resilience.

Mediating Pathways

- H7: Resilience will mediate the relationship between perceived stress and attitudes toward professional psychological help.
- H8a: Positive religious coping will mediate the relationship between perceived stress and attitudes toward professional psychological help.
- H8b: Negative religious coping will mediate the relationship between perceived stress and attitudes toward professional psychological help.

**Fig. 1:**
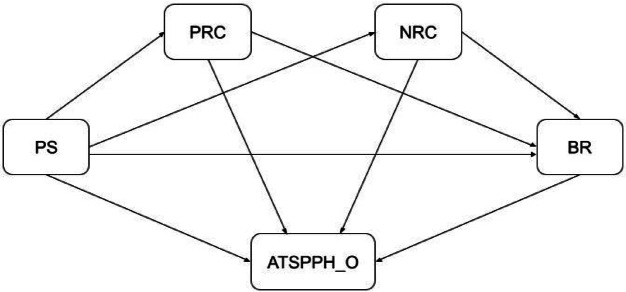
Proposed model.

This study proposes a conceptual framework that explores the interrelationships among four key psychological constructs: (1) perceived stress, (2) religious coping (positive and negative), (3) resilience, and (4) attitudes toward seeking professional psychological help, specifically within the context of Vietnamese university students. Guided by this theoretical reasoning, the proposed model (Figure 1) posits that perceived stress predicts both positive and negative religious coping, which in turn influence resilience and attitudes toward seeking professional psychological help. Moreover, resilience is expected to mediate the effects of perceived stress and religious coping on help-seeking attitudes. This framework thus integrates cognitive–behavioral and cultural–spiritual dimensions of coping to explain how stress translates into help-seeking intentions among Vietnamese university students.

Perceived stress is hypothesized to be positively associated with both positive and negative religious coping strategies (H1a, H1b), negatively associated with resilience (H2), and positively associated with attitudes toward seeking professional psychological help (H3). In turn, religious coping is posited to influence both help-seeking attitudes (H4a, H4b) and resilience levels (H6a, H6b), while resilience is expected to positively predict help-seeking attitudes (H5).

Furthermore, the model incorporates mediating mechanisms, whereby both religious coping and resilience are proposed to mediate the relationship between perceived stress and help-seeking attitudes (H7, H8a, H8b). Collectively, this conceptual model provides a theoretically grounded framework for examining the psychological and cultural processes that influence help-seeking behavior among university students in a collectivist, religiously influenced society.

## Methods

### Participants

Participants were undergraduate students recruited from multiple universities across Vietnam, such as institutions located in Ho Chi Minh City, Hanoi, Quang Ninh, and Da Nang. A network-based convenience sampling strategy was employed. The research team, including the principal investigator, student co-authors, and collaborating lecturers, disseminated an online questionnaire via Google Forms through their respective academic networks. In Ho Chi Minh City, student co-authors distributed the survey among their peers and class groups. In other provinces, lecturers within the researcher’s professional network facilitated access by sharing the survey with their students. Although random sampling was not feasible, deliberate efforts were made to ensure heterogeneity across geographic regions and academic disciplines, thereby enhancing sample diversity and mitigating selection bias.

Sample size determination followed the recommendation of Hair et al.^24^ for multivariate analyses, which suggests recruiting 5–10 participants per observed variable. Given that the study employed four psychometric instruments, each with a total of 40 observed items, the minimum required sample size ranged from 200 to 400 participants. A total of 564 responses were collected. After removing 148 responses due to incomplete data or uniform response patterns, the final analytic sample comprised 416 undergraduate students (263 females [63.2%], 153 males [36.8%]) aged between 18 and 25 years (M = 19.46, SD = 1.39).

Data collection was conducted online between November and December 2024. Participation was anonymous and voluntary, with no compensation provided. Informed consent was obtained electronically prior to beginning the survey, and participants were informed that they could discontinue participation at any time without penalty.

### Measurements

#### Perceived stress scale (PSS-10)

The Perceived Stress Scale (PSS), originally developed by Cohen, Kamarck, and Mermelstein^25^, is a self-report measure designed to assess the degree to which individuals appraise situations in their lives as stressful, particularly in terms of unpredictability, uncontrollability, and overload^25^. A widely used 10-item version (PSS-10) was later derived from the original 14-item version and has demonstrated satisfactory psychometric properties despite its brevity^26^. The original version of the PSS-10 has been shown internal consistency (Cronbach’s α = .78)^26^.

Each item is rated on a 5-point Likert scale ranging from 0 (never) to 4 (very often). Four positively worded items (Items 4, 5, 7, and 8) are reverse-coded before calculating a total score, with higher scores reflecting greater perceived stress. Example items include: “*In the last month, how often have you felt that you were unable to control the important things in your life?*” and “*In the last month, how often have you felt difficulties were piling up so high that you could not overcome them?*”^26^.

The Vietnamese version of the scale has been adapted and validated in previous research, demonstrating reliability with a Cronbach’s α of .80^27^. In the present study, internal consistency, as measured by Cronbach’s α, was acceptable (α =.69)^24^.

#### The attitude towards seeking professional psychological help scale—short form (ATSPPH- SF)

The Attitudes Toward Seeking Professional Psychological Help Scale - Short Form (ATSPPH-SF)^23^, a shortened version of the original 29-item scale by Fischer and Turner^29^, is a 10-item self-report instrument designed to assess individuals’ attitudes toward seeking professional psychological help. Each item is rated on a 4-point Likert scale ranging from 1 (disagree) to 4 (agree), with higher scores indicating more positive attitudes. The original version of the ATSPPH-SF has demonstrated internal consistency with a Cronbach’s α of .85^28^.

The scale consists of two subscales: Openness to seeking psychological help (Items 1, 3, 5, 6, and 7) and Value and Need in seeking professional psychological help (reverse-coded Items 2, 4, 8, 9, and 10). Example items include: “*I might want to have psychological counseling in the future*” and “*The idea of talking about problems with a psychologist strikes me as a poor way to get rid of emotional conflicts*” ^28^.

In this study, a Vietnamese-translated version of the ATSPPH-SF, rated on a 5-point Likert scale, was used^9^. The internal consistency, as measured by Cronbach’s α, was .83^9^. In the current sample, the ATSPPH-SF demonstrated acceptable overall reliability, with Cronbach’s alpha of .75^24^. When examined separately, the Openness to Seeking Professional Psychological Help subscale (ATSPPH-O) showed acceptable internal consistency (α = .78), while the Value and Need in Seeking Professional Psychological Help subscale (ATSPPH-N) exhibited lower reliability (α = .56)^24^.

#### Brief resilience scale (BRS)

The Brief Resilience Scale (BRS) is a 6-item self-report instrument developed by Smith et al.^30^ to assess individuals’ ability to recover or “bounce back” from stressful situations^30^. Each item is rated on a 5-point Likert scale ranging from 1 (strongly disagree) to 5 (strongly agree). Items 1, 3, and 5 are positively worded (e.g., “*I tend to bounce back quickly after hard times*”), while Items 2, 4, and 6 are negatively worded (e.g., “*I have a hard time making it through stressful events*”) and require reverse scoring. The final score is computed by averaging responses across all six items, with higher scores indicating greater resilience. The original validation study employed this scale and reported a satisfactory internal consistency, with a Cronbach’s alpha of .84^30^.

To ensure linguistic and cultural equivalence, both the Brief Resilience Scale (BRS) was adapted for Vietnamese use through a standardized forward–backward translation procedure^31,32^. Initially, the instruments were independently translated from English into Vietnamese by two native Vietnamese speakers who were fluent in English and familiar with psychological terminology. Discrepancies between the two versions were resolved through discussion and consensus. Subsequently, two independent bilingual experts, blinded to the original instruments, performed back-translations into English. The research team compared the back-translated versions with the originals to evaluate semantic, conceptual, and idiomatic equivalence. Minor discrepancies were reconciled to preserve the intended meaning of each item. The original item order was retained, and no additions or deletions were made. Although no qualitative pilot testing was conducted, the psychometric properties of the Vietnamese versions were evaluated post hoc using statistical validation techniques. Specifically, internal consistency was assessed using Cronbach’s alpha and partial least squares structural equation modeling (PLS-SEM), depending on distributional assumptions and model complexity. These analyses confirmed the factorial structure, reliability, and cultural appropriateness of the adapted instruments for use with Vietnamese university students. In the present study, the internal consistency of the BRS was acceptable (α = .69)^24^.

#### Brief religious coping scale (Brief RCOPE)

Brief Religious Coping Scale (Brief RCOPE), developed by Pargament et al.^33^, is a 14-item self-report instrument designed to assess religious coping strategies in response to stress^33^. Each item is rated on a 4-point Likert scale ranging from 0 (not at all) to 3 (a great deal). The scale comprises two subscales: positive religious coping (Items 1–7) and negative religious coping (Items 8–14). An example of a positive item is “*Looked for a stronger connection with God*,” while a negative item includes “*Wondered whether God had abandoned me*.” In the original validation study, internal consistency was satisfactory for both subscales, with Cronbach’s alpha of 0.90 for the positive subscale and 0.81 for the negative subscale^33^.

The Vietnamese version was adapted using the same standardized translation procedure described above. In the current study, the overall scale demonstrated excellent reliability, with a Cronbach’s alpha of 0.92^24^. Both subscales also showed strong internal consistency, with alpha values of 0.92 for Positive Religious Coping (PRC) and 0.91 for Negative Religious Coping (NRC)^24^.

### Data analysis

Data were encoded in Microsoft Excel and analyzed using the Statistical Package for Social Sciences (SPSS) version 28. Descriptive statistics were computed to summarize the frequency and percentage of categorical variables. Independent samples t-tests and one-way ANOVAs were conducted to examine differences in scale and subscale scores across demographic groups.

Structural equation modeling using SmartPLS 4.0 was employed to assess indicator reliability, construct reliability, convergent validity, and discriminant validity. Model fit was evaluated using variance inflation factors (VIFs), the standardized root mean square residual (SRMR), the coefficient of determination (R^2^), the effect size (f^2^), and the cross-validated redundancy (Q^2^). PLS-SEM was selected for its capacity to model complex variable relationships and its robustness with moderate sample sizes. Hypotheses were tested via partial least squares structural equation modeling (PLS-SEM) with 5000 bootstrap resamples to assess mediation effects.

## Results

### Demographic characteristics

The demographic characteristics of the participants are presented in Table 1. All participants were currently enrolled university students, with 140 (33.7%) majoring in humanities, 161 (38.7%) in education, and 115 (27.6%) in science, engineering, or technology disciplines. In terms of religious affiliation, 104 participants (25.0%) self-identified as religious, whereas the majority (n = 312; 75.0%) identified as non-religious. As noted by Pargament et al.^34^, religious coping measures—such as the RCOPE used in this study—encompass a broad range of spiritual beliefs and practices, including those held by individuals who do not formally affiliate with a religious tradition but may still engage in existential or spiritual coping (e.g., seeking meaning or comfort through non-institutional frameworks).

**Table 1:** The differences between demographic characteristics and the subfields.

Baseline characteristics	Total (n = 416)	PRC		NRC		BR		PS		ASTPPH-O	
	Frequency	Mean ± SD	p	Mean ± SD	p	Mean ± SD	p	Mean ± SD	p	Mean ± SD	p
*Gender*^*a*^											
Male	153 (36.8)	1.82 ± 0.83		1.42 ± 0.59		3.20 ± 0.73		2.05 ± 0.62		2.72 ± 0.76	
Female	263 (63.2)	1.89 ± 0.82		1.42 ± 0.66		3.08 ± 0.62		2.16 ± 0.54		2.85 ± 0.72	
Field of study^b^			< 0.05								
Humanities	140 (33.7)	2.04 ± 0.87		1.44 ± 0.60		3.12 ± 0.67		2.12 ± 0.61		2.71 ± 0.65	
Education	161 (38.7)	1.74 ± 0.77		1.36 ± 0.60		3.16 ± 0.61		2.11 ± 0.49		2.88 ± 0.73	
Science/Engineering/Technology	115 (27.6)	1.75 ± 0.71		1.02 ± 0.60		3.08 ± 0.71		2.14 ± 0.62		2.79 ± 0.83	
Religious Status^a^			< 0.001								
Religious	104 (25)	1.71 ± 0.74		1.39 ± 0.63		3.14 ± 0.69		2.10 ± 0.58		2.76 ± 0.76	
Non-religious	312 (75)	2.32 ± 0.90		1.50 ± 0.64		3.07 ± 0.59		2.17 ± 0.55		2.91 ± 0.65	
Sexual orientation^a^			< 0.05		< 0.05				< 0.05		
Heterosexual	293 (70.4)	1.81 ± 0.82		1.37 ± 0.60		3.12 ± 0.63		2.08 ± 0.57		2.82 ± 0.74	
LGBTQ + community	123 (29.6)	2.00 ± 0.80		1.56 ± 0.70		3.13 ± 0.73		2.22 ± 0.55		2.75 ± 0.73	
Self-harm behavior/thought^a^			< 0.05				< 0.001		< 0.001		< 0.001
No	249 (59.9)	1.79 ± 0.76		1.38 ± 0.59		3.27 ± 0.61		1.99 ± 0.54		2.90 ± 0.70	
Yes	167 (40.1)	1.99 ± 0.89		1.49 ± 0.68		2.91 ± 0.68		2.32 ± 0.57		2.66 ± 0.76	
Sources of support during difficulties^b^			< 0.05								
Self-help	40 (9.6)	1.72 ± 0.75		1.41 ± 0.75		3.22 ± 0.84		2.02 ± 0.69		2.79 ± 0.96	
Experts in psychology and mental health	36 (8.7)	2.20 ± 0.85		1.50 ± 0.77		3.06 ± 0.70		2.21 ± 0.70		2.58 ± 0.88	
Other sources	340 (81.7)	1.85 ± 0.82		1.42 ± 0.60		3.12 ± 0.63		2.12 ± 0.54		2.83 ± 0.68	

Values are presented as mean ± standard deviation (SD). a = Independent sample t-test, b = One-Way ANOVA. Abbreviations: PRC = Positive Religious Coping, NRC = Negative Religious Coping, BR = Resilience, PS = Perceived Stress, AS-O = Openness in Attitudes toward Professional Psychological Help.

Regarding sexual orientation, 293 participants (70.4%) identified as heterosexual, and 123 (29.6%) identified as members of the LGBTQ + community. Concerning mental health experiences, 167 participants (40.1%) reported a history of self-harmed thoughts or behaviors, while 249 (59.9%) reported no such experiences. When asked about sources of support during personal difficulties, 40 participants (9.6%) reported relying on self-help strategies, 36 (8.7%) sought assistance from mental health professionals, and the majority (n = 340; 81.7%) reported turning to other sources, such as friends, family members, or substance use.

### Preliminary analysis

Given the cross-sectional design and single-source data collection, we assessed multivariate normality and common method bias (CMB) to ensure the validity of the constructs. Multivariate normality was examined using Mardia’s multivariate skewness and kurtosis statistics.

The results revealed significant deviations from normality, with Mardia’s skewness (b = 305.97, z = 21213.63, *p* < 0.001) and kurtosis (b = 1984.10, z = 53.50, *p* < 0.001), indicating non-normal multivariate distribution. However, these results are not uncommon in large samples (N = 416), as Mardia’s tests are known to be highly sensitive to sample size, frequently detecting significant deviations even in cases of minimal distributional asymmetry^35,36^. Despite the multivariate non-normality, all univariate skewness and kurtosis values were within acceptable thresholds (± 3 for skewness and ± 10 for kurtosis), following the criteria proposed by Kline^37^. Given that partial least squares structural equation modeling (PLS-SEM) does not require multivariate normality, these deviations were not considered problematic for subsequent analyses.

CMB occurs when data for both independent and dependent variables are obtained from the same respondent using the same measurement context, potentially inflating the observed associations among variables^38^. To address potential CMB, Harman’s single-factor test was employed. The results indicated that a single factor accounted for 20.30% of the total variance, well below the 50% threshold, suggesting that CMB was not a major concern^39^. Additionally, full collinearity variance inflation factors (VIFs) for all constructs were examined. All VIF values were below the conservative cutoff of 5.0, further confirming the absence of substantial common method bias^39^.

### Comparison test

As shown in Table 1, independent sample *T*-tests were conducted to assess mean differences across demographic groups, including gender, religious status, sexual orientation, and self-harm history (see Table 1). No significant gender differences were found across the primary constructs (*p* > 0.05). Participants identifying as religious (n = 104) reported significantly higher Positive Religious Coping (PRC) (M = 2.32, SD = 0.90) than those identifying as non-religious (n = 312; M = 1.71, SD = 0.74), t(151.52) =  − 6.23, *p* < 0.001. LGBTQ + participants (n = 123) reported higher Positive Religious Coping (PRC) (M = 2.00, SD = 0.80) than heterosexual participants (n = 293; M = 1.81, SD = 0.83), t(414) = 2.17, *p* = 0.031. They also reported higher Negative Religious Coping (NRC) (M = 1.56, SD = 0.70 vs. M = 1.37, SD = 0.60), t(≈199) = 2.60, *p* = 0.010, and higher Perceived Stress (PS) (M = 2.22, SD = 0.55 vs. M = 2.08, SD = 0.58), t(414) = 2.26, *p* = 0.024. No significant group differences were found for Resilience (BR) or Openness in Attitudes toward Professional Psychological Help (ATSPPH-O) (ps > 0.05). Participants with a history of self-harm (n = 167) reported significantly higher positive religious coping (PRC) (M = 1.99, SD = 0.89) compared to those without such history (n = 249; M = 1.79, SD = 0.76), t(414) = 2.44, *p* = 0.015, as well as higher perceived stress (PS) (M = 2.32, SD = 0.57 vs. M = 1.99, SD = 0.54), t(414) = 6.15, *p* < 0.001. Meanwhile, they reported lower resilience (BR) (M = 2.91, SD = 0.68 vs. M = 3.27, SD = 0.61), t(414) =  − 5.68, *p* < 0.001, and lower openness in attitudes toward professional psychological help (ATSPPH-O) (M = 2.66, SD = 0.76 vs. M = 2.90, SD = 0.70), t(414) =  − 3.21, *p* = 0.001. No significant group difference was found for negative religious coping (NRC) (*p* > 0.05).

A one-way analysis of variance (ANOVA) was conducted to examine differences across academic majors and sources of psychological support. For academic majors, a significant group difference was found in positive religious coping (PRC), F(2, 413) = 5.30, *p* = 0.005. Post hoc analyses using Tukey’s HSD indicated that students majoring in the humanities (M = 2.04, SD = 0.87) reported significantly higher PRC than those in education (M = 1.74, SD = 0.77; *p* = 0.004). No significant differences were observed between humanities and science/engineering/technology majors (*p* = 0.079), nor between education and science/engineering/technology majors (*p* = 0.713). No significant group differences were found for negative religious coping (NRC), resilience (BR), perceived stress (PS), or openness to seeking professional psychological help (ATSPPH-O) (ps > 0.05). Regarding sources of support during difficulties, a significant group difference was found for positive religious coping (PRC), F(2, 413) = 3.71, *p* = 0.025. Tukey’s HSD indicated that participants who sought support from experts in psychology and mental health (M = 2.20, SD = 0.85) reported higher PRC than those relying on self-help (M = 1.72, SD = 0.75; *p* = 0.029) and those using other sources (M = 1.85, SD = 0.82; *p* = 0.040). No significant group differences were observed for negative religious coping (NRC), resilience (BR), perceived stress (PS), or openness to seeking professional psychological help (ATSPPH-O) (ps > 0.05).

### Measurement model

The measurement model was assessed using partial least squares structural equation modeling (PLS-SEM), implemented via SmartPLS 4.0, to evaluate construct reliability and validity. Although this study employed previously validated scales, all outer loadings were reassessed to evaluate item-level reliability within the Vietnamese context. As shown in Table 2, most standardized factor loadings exceeded the recommended threshold of 0.70^40^. However, a few reverse-coded items in the Perceived Stress (e.g., PS7 = –0.002) and Resilience (e.g., BR3 = 0.412) constructs demonstrated lower loadings. These items were retained because their exclusion did not meaningfully improve model fit indices or convergent validity, and they were theoretically important for preserving content validity and mitigating acquiescence bias.

**Table 2:** Factor Analysis and Reliability.

Research constructs	Factors	Factor loadings	Eigen-value	Percentage of variance explained	Item to total correlation	Cronbach’s α
RC	PRC	0.76–0.86	7.58	54.12	0.63–0.82	0.92
	NRC	0.64–0.87	2.02	14.43	0.55–0.82	0.91
PS	–	-0.002–0.79	2.93	48.91	0.42–0.65	0.69
BR	–	0.41–0.77	3.83	63.82	0.34–0.49	0.69
ATSPPH-O	–	0.56–0.85	2.72	54.36	0.41–0.69	0.78

Factor loadings represent the range of values for each construct. Cronbach’s α indicates internal consistency reliability. Abbreviations: RC = Religious Coping, BR = Resilience, PS = Perceived Stress, ATSPPH = Attitudes Toward Professional Psychological Help.

This phenomenon is consistent with findings from cross-cultural research, where reverse-worded items frequently exhibit low or even negative loadings. Such issues are often attributed to reverse-wording effects, especially when data are collected via online self-report forms in non-English-speaking populations^41^. In these contexts, participants may experience item verification difficulty, leading to “*misresponse*” — that is, selecting responses on the same side of the neutral point for both reversed and non-reversed items. This effect is more likely to occur when task complexity increases due to the cognitive operations required to interpret reversed semantics, particularly in digital environments or among respondents with lower verbal comprehension.

Table 3 presents the Average Variance Extracted (AVE), Composite Reliability (CR), and Cronbach’s alpha for each construct. While the AVE values for Perceived Stress (AVE = 0.30) and Resilience (AVE = 0.39) did not reach the 0.50 benchmark suggested by Fornell and Larcker^42^, all constructs demonstrated satisfactory composite reliability (CR > 0.70)^42^. According to Hair et al.^40^, convergent validity can still be considered adequate when CR is high, even if AVE falls below 0.50. This supports the robustness of the measurement model in the current context.

**Table 3:** Measurement Scale Items of This Study.

Construct	AVE	CR	R2
PS	0.30	0.77	–
PRC	0.68	0.94	0.03
NRC	0.67	0.93	0.02
BR	0.39	0.78	0.30
ATSPPH-O	0.54	0.85	0.15

AVE = Average Variance Extracted, CR = Composite Reliability, Cronbach’s alpha indicates internal consistency reliability, R^2^ = Coefficient of Determination. A dash (–) indicates that data were not reported.

In terms of discriminant validity, the Heterotrait-Monotrait ratio of correlations (HTMT) was used. All HTMT values were below the conservative threshold of .85 ^43^, ranging from .09 to .73. The highest observed HTMT value was between Perceived Stress and Resilience (HTMT = .73), which remains within acceptable limits, supporting the discriminant validity of all constructs. This suggests that each construct is empirically distinct from the others in the model.

The coefficient of determination (R^2^) for endogenous latent variables indicates the proportion of variance explained by the model^40^. PRC (R^2^ = .03), NRC (R^2^ = .02), BR (R^2^ = .30), and ATSPPH-O (R^2^ = .15) exhibited mild to moderate explanatory power, suggesting that the model accounts for 2.0–30.3% of the variance in these constructs, though predictive strength varies^37^. To evaluate the model’s predictive relevance, the blindfolding procedure was conducted. The cross-validated redundancy Q^2^ values for all endogenous constructs were greater than zero (e.g., ATSPPH_O = .09; BR = .27; NRC = .01; PRC = .02), suggesting that the model has small but positive predictive relevance^40^. The Q^2^ value for PS was not computed since it was not modeled as an endogenous construct.

### Structural model

The model fit was evaluated using the Standardized Root Mean Square Residual (SRMR), which is considered the most appropriate fit index for PLS-SEM^40,43^. The SRMR value for the saturated model was 0.078, which is below the recommended cut-off of 0.08, indicating an acceptable fit. However, the estimated model yielded an SRMR of 0.137, suggesting potential areas for refinement. Additional discrepancy measures, d_ULS and d_G, were also examined and fell within the bootstrapped confidence intervals, supporting model adequacy. Although the Normed Fit Index (NFI = 0.73) was below the commonly accepted threshold, PLS-SEM emphasizes predictive accuracy over exact model fit. Therefore, SRMR remains the most reliable index in this context.

Table 4 illustrates the relationship between the research constructs. To address the inflation of Type I error due to testing 12 hypotheses, we applied the False Discovery Rate (FDR) correction using the Benjamini-Hochberg procedure with a significance threshold of q = 0.05. The findings indicate that perceived stress (PS) is positively associated with positive religious coping (PRC) (H1a: β = 0.16, t = 2.94, p_adjusted = 0.008, 95% CI [0.09, 0.27]) and negative religious coping (NRC) (H1b: β = 0.14, t = 2.98, p_adjusted = 0.008, 95% CI [0.07, 0.22]), supporting hypotheses H1a and H1b. PS is negatively associated with resilience (BR) (H2: β = −0.54, t = 11.96, p_adjusted < 0.001, 95% CI [−0.62, −0.47]), supporting H2. Additionally, PS exerts a strong positive correlation with openness in attitudes toward seeking professional psychological help (ATSPPH-O) (H3: β = 0.25, t = 4.15, p_adjusted < 0.001, 95% CI [0.16, 0.35]), confirming H3. Positive religious coping (PRC) demonstrates a robust positive effect on ATSPPH-O (H4a: β = 0.27, t = 4.72, p_adjusted < 0.001, 95% CI [0.17, 0.36]), supporting H4a. In contrast, the effect of negative religious coping (NRC) on ATSPPH-O (H4b: β = −0.15, t = 2.24, p_adjusted = 0.058, 95% CI [−0.26, −0.04]) was not statistically significant after FDR correction, failing to support H4b. The effect of BR on ATSPPH_O was not statistically significant (H5: β = −0.09, t = 1.29, p_adjusted = 0.202, 95% CI [−0.20, 0.02]), failing to support H5. Similarly, the effect of PRC on BR lacked statistical significance (H6a: β = 0.07, t = 1.41, p_adjusted = 0.202, 95% CI [−0.01, 0.16]), failing to support H6a. The effect of NRC on BR (H6b: β = −0.11, t = 2.11, p_adjusted = 0.060, 95% CI [−0.19, −0.03]) was also not statistically significant after FDR correction, failing to support H6b. The indirect effect of PS on ATSPPH_O via BR was not statistically significant (H7: β = 0.05, t = 1.26, p_adjusted = 0.202, 95% CI [−0.01, 0.11]), failing to support H7.

**Table 4:** Evaluation of Structural Model and Hypothesis Testing.

Hyp	Path	Standardize Estimate	t-value	95% CI	p-value	Adjusted p-value (FDR)	Hypothesis testing
H1a	PS → PRC	0.16	2.94	[0.09, 0.27]	0.003	0.008	Significant
H1b	PS → NRC	0.14	2.98	[0.07, 0.22]	0.002	0.008	Significant
H2	PS → BR	-0.54	11.96	[−0.62, -0.47]	< 0.001	< 0.001	Significant
H3	PS → ATSPPH-O	0.25	4.15	[0.16, 0.35]	< 0.001	< 0.001	Significant
H4a	PRC → ATSPPH-O	0.27	4.72	[0.17, 0.36]	< 0.001	< 0.001	Significant
H4b	NRC → ATSPPH-O	-0.15	2.24	[−0.26, -0.04]	0.024	0.058	Not Significant
H5	BR → ATSPPH-O	-0.09	1.29	[−0.20, 0.02]	0.194	0.202	Not Significant
H6a	PRC → BR	0.07	1.41	[−0.01, 0.16]	0.160	0.202	Not Significant
H6b	NRC → BR	-0.11	2.11	[-0.19, -0.03]	0.036	0.060	Not Significant
H7	PS → BR → ATSPPH-O	0.05	1.26	[−0.01, 0.11]	0.202	0.202	Not Significant
H8a	PS → PRC → ATSPPH-O	0.04	2.61	[0.02, 0.07]	0.009	0.019	Significant
H8b	PS → NRC → ATSPPH-O	−0.02	1.72	[−0.04, 0.00]	0.083	0.125	Not Significant

*Standardized estimate represents the path coefficient in the structural model. t-value indicates statistical significance. 95% CI = 95% Confidence Interval.

The study identifies a significant positive indirect effect of PS on ATSPPH_O through PRC (H8a: β = 0.04, t = 2.61, p_adjusted = 0.019, 95% CI [0.02, 0.07]), supporting H8a. However, the indirect effect via NRC (H8b: β = −0.02, t = 1.72, p_adjusted = 0.125, 95% CI [−0.04, 0.00]) was not statistically significant, failing to support H8b.

**Fig. 2:**
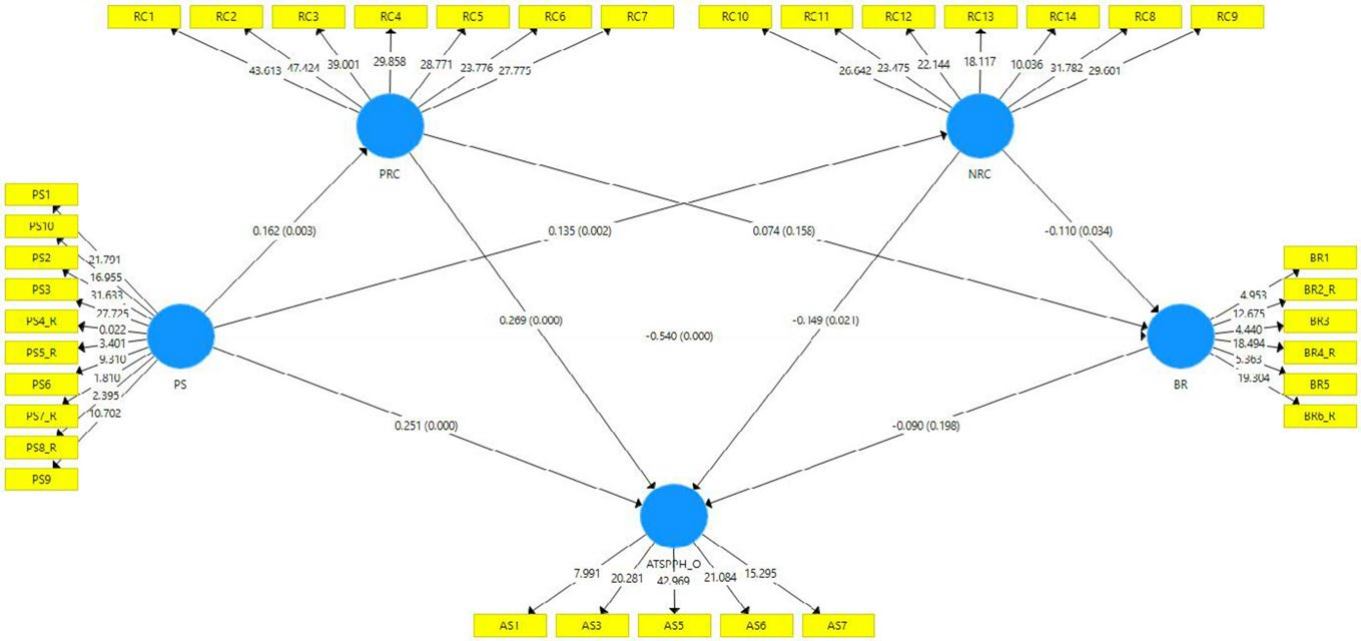
Model of PLS-SEM.

Path coefficients were estimated using 5000-sample bootstrapping. *p*-values were adjusted for multiple testing using the Benjamini–Hochberg false discovery rate correction (q = 0.05). Table 4 reports adjusted p-values used for statistical inferences, which may differ from the unadjusted *p*-values shown in Figure 2 for illustration purposes.

The results indicate that perceived stress has a significant influence on both resilience and attitudes toward seeking professional psychological help, with a direct positive association observed in the latter. Additionally, perceived stress has an indirect effect on help-seeking attitudes through positive religious coping. In contrast, the effects of negative religious coping were not statistically significant after adjusting for the FDR. Hypotheses H4b, H5, H6a, H6b, H7, and H8b were not supported.

## Discussion

This study advances our understanding of the psychological mechanisms underlying help-seeking attitudes among Vietnamese university students by integrating perceived stress, religious coping, and resilience within a theoretically grounded model. Based on empirical results and comparative literature analysis, the following key insights emerge.

The finding that perceived stress significantly predicted both positive and negative religious coping aligns with the theoretical postulates of the Stress and Coping Theory^19^ and prior empirical study^1^. Students experiencing elevated stress may turn to religious coping as a dual-function mechanism: to regulate emotional responses (positive coping) or to externalize distress through spiritual struggle (negative coping), while positive religious coping shows no clear association with stress levels^13,14,45^. Our findings corroborate this duality, which has been documented in cross-cultural samples including emerging adults in the U.S.^44^ and Portugal^12^, as well as in Vietnamese^9^ and Chinese students^2,17^. Notably, the link between stress and positive religious coping suggests students seek comfort through prayer, communal faith, or a sense of divine control, consistent with patterns observed in religiously committed youth^46^. Conversely, the presence of negative religious coping (NRC), such as spiritual discontent or punitive appraisals, aligns with prior findings that unresolved stress may provoke theological conflict, particularly in collectivist or authoritarian cultural milieus^13,14,44^. The current findings are consistent with recent Vietnamese studies emphasizing the adaptive and motivational functions of religiosity under stress. Huynh et al.^20^ found that intrinsic religiosity among Vietnamese believers was associated with higher gratitude and happiness, with gratitude mediating this relationship^20^. The results suggest that religiosity primarily strengthens internal emotional regulation through gratitude and positive effect rather than prompting behavioral change toward external psychological help. In line with this, the present study indicates that while positive religious coping enhances resilience, it does not directly increase openness to professional psychological services^20^. Similarly, Nguyen et al.^21^, although focusing on entrepreneurial motivation rather than mental health, demonstrated that intrinsic religiosity indirectly enhanced entrepreneurial motivation through self-efficacy and stress tolerance^21^. This suggests that religiosity serves as an internal psychological resource, enhancing individuals’ adaptive capacity in the face of stress. Such a mechanism parallels the present study’s findings, in which positive religious coping was associated with greater resilience but did not translate into higher levels of professional help-seeking. Together, these results suggest that in Vietnamese culture, religiosity primarily functions as an inner stabilizer of emotion and morality—enhancing psychological endurance and meaning-making—rather than as a behavioral determinant guiding the use of formal psychological services.

Positive religious coping (PRC) to resilience was not statistically significant; the weak positive tendency is not theoretically consistent with previous findings, which suggest that religious coping may enhance adaptive psychological functioning^12,14^. This aligns with Pargament’s conceptualization of benevolent religious reappraisals and seeking religious support as meaning-making mechanisms that bolster inner strength^13^. PRC showed a significant positive association with help-seeking attitudes, indicating that students who engage in positive forms of religious coping may be more open to professional psychological assistance. In contrast to previous studies, cultural nuances in which religious youth turn to spiritual communities or informal sources (e.g., family, peers) rather than professional services^4,5,8^. Prior studies in Asian settings (e.g., South Korea^48^, Vietnam^4^, and Taiwan^8^) have shown a strong preference for informal or spiritual forms of support. This finding suggests that among Vietnamese students, PRC may facilitate openness toward professional psychological services, consistent with the notion that adaptive faith-based coping enhances receptivity to mental health support. However, this association should be understood within Vietnam’s moral–religious context, where spiritual values intertwine with filial piety and self-discipline. As noted by Nguyen et al.^22^, the cultural emphasis on emotional restraint and moral self-control encourages individuals to strike a balance between personal endurance and selective openness to external help^22^. In such settings, the PRC may act both as a psychological buffer—providing internal stability through prayer or reflection—and as a bridge that legitimizes seeking professional aid when personal coping mechanisms are insufficient. Similarly, Huynh et al.^23^ observed that religious beliefs mediate the link between well-being and fear, suggesting that faith serves not merely as an inward cognitive schema but also as a framework that shapes adaptive behavioral choices^23^. Therefore, in the Vietnamese cultural milieu, PRC appears to integrate inner meaning-making with emerging willingness to engage in formal mental health services.

Although negative religious coping (NRC) initially showed a negative association with attitudes toward seeking psychological help, this effect did not remain significant after controlling for multiple comparisons using the FDR correction. This finding contrasts with prior studies suggesting that spiritual struggle often correlates with poorer mental health outcomes and avoidance of professional services^13,44^. Similarly, although the direct path from resilience to help-seeking attitudes was not statistically significant, the coefficient indicated a weak negative tendency, consistent with research showing that more self-reliant or emotionally resilient students may perceive a lesser need for professional intervention^6,16,45^. In line with findings from a large Italian sample^7^, where higher distress predicted more coping and lower stigma facilitated help-seeking, the present results refine this pattern by suggesting that resilience may act as a functional substitute for professional reliance, particularly in contexts where help-seeking remains stigmatized^4,10,47^. While NRC was initially found to negatively predict resilience, this relationship did not retain statistical significance after controlling multiple comparisons using the FDR correction. This attenuation may reflect the nuanced role of religiosity in Vietnamese contexts, where intrinsic faith and gratitude often foster well-being rather than maladaptive coping^20,23^. Moreover, moral self-discipline and filial piety emphasize emotional restraint and self-regulation, which may buffer the detrimental effects of spiritual struggle on resilience^21,22^.

The subgroup analysis revealed that LGBTQ + students reported significantly higher positive and negative religious coping and higher perceived stress than heterosexual peers, while no significant differences were found in resilience or openness to professional psychological help. While the elevated perceived stress among LGBTQ + students is consistent with prior evidence on minority stress, the lack of group differences in help-seeking openness contrasts with earlier findings that emphasize structural and attitudinal barriers to formal mental health services among sexual minority populations^5,6,15,46^.

Furthermore, students with religious affiliation demonstrated stronger engagement in positive religious coping compared with their non-religious counterparts. This pattern aligns with the conceptualization of positive religious coping as reliance on faith-based and meaning-oriented resources and may indicate that religious students more readily draw on spiritual beliefs, practices, and community-based support when managing stressors. Such reliance on informal and culturally embedded coping resources is consistent with prior findings among Vietnamese and East Asian college students, who tend to prioritize family, close relationships, and belief systems over formal mental health services^4,8^.

Humanities majors showed higher positive religious coping (PRC) than students in education, echoing prior evidence that exposure to psychosocial and cultural knowledge may foster reflective coping and value-based self-regulation.^10,11^.

Students who reported self-harmed thoughts or behaviors exhibited significantly higher levels of positive religious coping, alongside greater perceived stress, but lower resilience and openness toward professional psychological help. This pattern suggests that self-harming individuals may turn to internal or spiritual forms of coping as an immediate emotional regulation strategy while remaining less inclined to seek professional assistance, potentially due to stigma and cultural expectations of emotional restraint. The elevated stress and diminished resilience observed in this group are consistent with prior evidence linking self-harm vulnerability with limited adaptive coping resources and reduced engagement with formal mental health services among Asian youth^9–11,46^. Such tendencies underscore the role of collectivist norms—particularly concerns about family reputation and social harmony, discouraging disclosure and help-seeking, thereby reinforcing reliance on personal or spiritually oriented coping mechanisms over therapeutic engagement^4,8,10,11^.

Students who sought help from experts in psychology and mental health reported significantly higher levels of positive religious coping than those relying on self-help or other informal sources. This pattern suggests that professional guidance may reinforce adaptive forms of religious coping by integrating cognitive reframing and meaning-making processes into therapeutic interactions^4,8^. Conversely, students who depend primarily on self-reliance or informal networks may engage in less structured or culturally constrained coping, consistent with prior findings that stigma, family expectations, and limited campus-based mental health services deter formal help-seeking among Vietnamese youth^9–11,46^. Similar observations have been noted in other collectivist settings, where professional and spiritual coping often coexist as complementary pathways for emotional regulation rather than mutually exclusive alternatives^6,8^.

### Limitations

First, the cross-sectional design constrains causal inference and limits the ability to observe temporal changes in coping and help-seeking processes. Longitudinal or daily diary designs may more effectively capture dynamic fluctuations in these relationships. Second, reliance on self-report measures introduces potential biases, including social desirability effects and response fatigue. Future research employing mixed-method approaches, integrating surveys with interviews or digital behavior tracking—would enhance ecological validity. Third, although the sample included gender and sexual-identity diversity, participants were drawn solely from urban university settings, which restricts generalizability. Future studies should include rural and vocational students to represent Vietnam’s educational landscape more comprehensively. Fourth, while validated instruments were used, certain scales demonstrated suboptimal internal consistency (e.g., Perceived Stress Scale and Brief Resilience Scale). Low factor loadings and an NFI below 0.80 indicate the need for refinement or localization of specific items. Fifth, key cultural variables—such as stigma, family expectations, and religious commitment—were excluded due to the absence of validated Vietnamese measures. Future research should therefore develop culturally grounded instruments to examine these latent influences. Finally, the limited predictive power of positive religious coping and resilience underscores the importance of broader conceptual models that incorporate digital coping mechanisms (e.g., online peer forums), peer influences, and health-service accessibility. Programs designed to reduce stigma and promote psychological literacy remain crucial for bridging the gap between emotional regulation and professional service utilization.

### Implications

#### Theoretical implications

This study advances a culturally contextualized understanding of university students’ help-seeking behaviors by modeling how perceived stress activates both adaptive and maladaptive coping mechanisms. The concurrent engagement in positive religious coping (e.g., prayer, spiritual support) and negative religious coping (e.g., spiritual discontent, punitive beliefs) illustrates the complex interplay between emotional regulation and behavioral activation within stress-response processes. Although both positive religious coping and resilience functioned as buffers against psychological distress, neither construct directly predicted professional help-seeking, implying that these factors may operate primarily as internal self-regulatory resources rather than as behavioral catalysts. Furthermore, the negative association between resilience and help-seeking attitudes reveals a potential paradox: students who perceive themselves as emotionally strong or self-reliant may experience a reduced motivation to seek professional assistance, particularly in sociocultural environments where psychological services are stigmatized. This finding suggests that psychological strength alone does not necessarily translate into proactive help-seeking behavior. Cultural mediators—including social stigma, family expectations, and norms of emotional restraint—may act as latent suppressors that inhibit behavioral expression even under psychological strain. Collectively, these findings highlight the need for refined, culturally informed models that integrate interconnected psychosocial and cultural constructs, rather than relying solely on linear, cause-and-effect frameworks derived from Western paradigms.

#### Practical implications

The findings of this study underscore urgent structural needs in mental health service delivery within Vietnamese higher education. A primary recommendation is the institutionalization of mandatory on-campus psychological counseling centers across all universities. These centers should be fully staffed by qualified mental health professionals and recognized as formal institutional units rather than auxiliary or temporary services. Embedding psychological support as a core component of student services could help reduce stigma, normalize help-seeking, and improve overall mental health outcomes.

Given the prevalence of spiritual coping strategies among Vietnamese students, universities are also encouraged to establish inclusive spaces for religious and spiritual practices, such as prayer rooms or meditation areas. Recognizing such practices as legitimate forms of emotional regulation—without privileging any specific belief system—would foster a psychologically safe and inclusive campus environment for students of diverse faiths and worldviews.

Furthermore, mental health literacy programs should be systematically integrated across academic disciplines and made accessible to all students, not solely those in psychology or health-related fields. These initiatives can be incorporated into orientation sessions, general education curricula, or campus-wide awareness campaigns. By enhancing students’ understanding of psychological well-being, adaptive coping mechanisms, and available support services, universities can promote earlier intervention and reduce reliance on informal or solitary coping strategies.

Finally, collaboration between mental health professionals and trusted spiritual or community leaders may serve as a culturally sensitive bridge for students hesitant to engage with formal services. Providing these individuals with training in psychological first aid and referral procedures would facilitate appropriate care access while maintaining respect for cultural norms and personal belief systems. Collectively, these practical measures would contribute to the creation of a campus-wide mental health ecosystem that is both responsive and culturally attuned to the diverse needs of the student population.

## Conclusion

This study advances a culturally informed understanding of professional psychological help-seeking among Vietnamese university students by clarifying the roles of perceived stress, religious coping, and resilience. The findings indicate that perceived stress is directly associated with greater openness to professional psychological help and indirectly related through positive religious coping, underscoring the dual function of stress as both a catalyst for external support and a trigger for internal, meaning-based coping. In contrast, negative religious coping and resilience did not significantly predict help-seeking attitudes, suggesting that these constructs primarily serve emotional regulatory functions rather than facilitating formal help-seeking behavior. Subgroup analyses further revealed heterogeneity in help-seeking pathways: LGBTQ + students reported elevated stress and greater use of both positive and negative religious coping without differences in resilience or help-seeking openness, whereas students with a history of self-harm exhibited higher stress and positive religious coping alongside lower resilience and reduced openness to professional help. Together, these findings highlight the importance of integrating cultural, spiritual, and psychological factors in mental-health interventions and addressing internalized stigma and culturally embedded norms that shape students’ engagement with professional mental-health services.

## Data Availability

The dataset generated during and/or analyzed in the current study will be made available from VLTC upon reasonable request, once the authors have fully exploited the dataset. No supplementary information is provided with this manuscript.
